# Integrated Healthcare Systems Response Strategies Based on the Luohu Model During the COVID-19 Epidemic in Shenzhen, China

**DOI:** 10.5334/ijic.5628

**Published:** 2021-02-02

**Authors:** Fangfang Gong, Guangyu Hu, Hanqun Lin, Xizhuo Sun, Wenxin Wang

**Affiliations:** 1 Shenzhen Luohu Hospital Group, Shenzhen, China; 2 Institute of Medical Information/Center for Health Policy and Management, Chinese Academy of Medical Sciences and Peking Union Medical College, Beijing, China; 3 Department of Public Administration, Law School, Shantou University, Shan-Tou, China

**Keywords:** integrated healthcare system, COVID-19, epidemic, preparedness, health emergency

## Abstract

COVID-19 has affected primary health-care delivery in metropolitan areas. An integrated health-care system offers advantages in response to the community outbreak and transmission of highly infectious diseases. On the basis of practitioner experience with a pioneering integrated health-care system in Shenzhen, China, this article presents the following effective strategies in response to the epidemic: (1) enhance the public workforce in primary health care; (2) integrate resources to allow regional sharing and efficient use; (3) employ teams centered on general practitioners for community containment; and (4) adopt e-health and telemedicine for health-care delivery. An integrated health-care system is usually very specific to a particular regional context; however, the core strategies and mechanisms based on the Luohu model can contribute to improving the public health capacity in emergency responses; they can transform health-care delivery in the COVID-19 epidemic. The experience in Shenzhen may help other cities in enhancing and coordinating the preparedness of their health-care systems in dealing with future public health emergencies.

## INTRODUCTION

The recent coronavirus disease (COVID-19) epidemic has led to a surge in demand for health-care services in urban areas; just as in Wuhan, China, worldwide demand has overwhelmed the response capacities of the centralized hospital and dispersed health-care systems [1]. In the early stage of the epidemic, that posed an unprecedented challenge for Wuhan and other metropolitan centers in attempts to contain local transmission. China’s primary health-care system is responsible for preventing and managing emerging infectious diseases, such as COVID-19. However, there remain substantial discrepancies in the quality of care between primary health-care institutions and hospitals [2]. Thus, policy makers need to identify and implement careful, coordinated solutions in response to the COVID-19 epidemic; they also have to reinforce the responsibility of the primary health-care system in preventing and managing infectious diseases.

In 2016, the World Health Organization, World Bank, and Chinese government jointly published a report, which proposed enhancing China’s health-care system through a person-centered integrated care model [3]. The aim with this health-care service model is to provide people with a continuum of care: the range is from health promotion and preventive care to treatment services throughout a person’s life; it is coordinated across various type of health-care facilities according to a patient’s needs. Public health is a vital part of this system. And people—not necessarily patients—are the prime focus [4]. Integrated health-care systems have potential advantages in response to community outbreaks of highly infectious diseases (such as COVID-19) and their transmission. Those advantages may include the following: primary care for risk stratification with first contact support; multidisciplinary teams for high-risk patients; vertical and horizontal integration of different types of providers and care; and integrated e-health across facilities and services.

**Figure 1:**
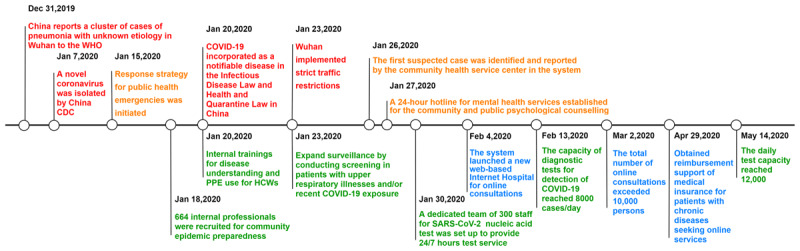
Timeline of the Luohu integrated health-care system response during the coronavirus disease (COVID-19) outbreak in China. Red lettering indicates important time points in the fight against the epidemic in China; yellow lettering indicates the key responses of the Luohu Hospital Group; green lettering signifies unified allocation of resources in the Luohu Hospital Group; blue lettering denotes Internet Hospital work in the Luohu Hospital Group.

In this paper, we present the response strategies and experiences of an integrated health-care system in Shenzhen, a city in eastern China, during the COVID-19 pandemic. The integrated service delivery model on which the system is based is known as the Luohu model [5]. It was introduced and practiced in the Luohu district of Shenzhen in 2015. The Luohu Integrated Health-Care System (Luohu Hospital Group) was established as an integrated organization and independent legal entity; it comprises five hospitals, 23 community health service institutions, six resource-sharing centers, six administrative centers, and an institute of precision medicine. This system was activated immediately after the first COVID-19 outbreak in January 2020 (***Figure 1***). During the outbreak in China, community-based integrated health-care systems have become the primary means of effective community transmission containment. Those response strategies have played an important role in limiting community transmission and slowing the outbreak.

## COVID-19 RESPONSE STRATEGIES IN THE LUOHU HOSPITAL GROUP

- **Enhancing public health workforce in primary health care**

It is recognized that community health workers play a pivotal role in fighting the COVID-19 pandemic [6]. An inadequate public health workforce is a major challenge to delivering high-quality basic public health services, such as a functional response to emerging infectious diseases in China and other low- and middle-income countries [7].

The Luohu Hospital Group required all its 23 community health service institutions to recruit and equip professional public health personnel before those institutions were fully qualified and acknowledged by the group. The group developed a unified career development policy for public health personnel so that they can better assist institutions in providing primary health care and basic public health services for the community. The ratio of public health professionals to general practitioners in the group increased from 1:6.24 in 2015 to 1:3.97 in 2019. The increase in professional public health personnel has improved the Luohu Hospital Group’s overall ability to deliver professional public health services to the community.

The Luohu Hospital Group has also cooperated with the local Center for Disease Control and Prevention (CDC). The group has employed external public health experts from the CDC to participate in the routine work of community health service institutions. Those experts are responsible for providing professional public health training to community health workers; the experts offer on-site assistance in dealing with public health emergencies in communities. This collaboration with the local CDC has improved the capability of community health workers to cope with public health issues in the integrated health-care system. Moreover, the cooperation constitutes an important foundation for the system response to public health emergencies, such as COVID-19. These practices have enhanced collaboration between the Luohu Hospital Group and the local CDC as well as assisting in disease control and efforts to prevent epidemics.

Since the integrated health-care system was established in 2015, community health service institutions in the Luohu Hospital Group have made ongoing efforts to increase their ability to undertake infectious disease monitoring. On January 20, 2020, China’s central government classified COVID-19 as a Class B infectious disease (i.e., any medical institution that diagnoses a suspected case has to report it to the government); the government adopted Class A infectious disease management measures against COVID-19. Less than a week later (January 26), one community health service institution in the Luohu Hospital Group reported its first suspected case of COVID-19 in the community to the local CDC; subsequently, the case was confirmed as COVID-19.

At present, the community health service institutions in the Luohu Hospital Group make most of the initial contacts with patients from the community. The number of outpatient visits to community health service institutions in 2020-Q1 accounted for 43.49% of the group’s total outpatient visits: in 2020-Q1, the number of group outpatient visits was 531,600; that to community health service institutions was 231,300. Through the group’s competent public health workforce, the community health service institutions have been able to conduct COVID-19 screening, triage, and monitoring and reporting for community patients. Thus, the health-care system was able to deal with the COVID-19 epidemic in an organized fashion.

- **Integrated resources permit regional sharing and efficient use**

The established resource-sharing centers and administrative centers in the Luohu Hospital Group are two core areas toward achieving an integrated health-care system. Using the synchronized integration of the group’s health-care and management resources, such an integrated health-care system supports a unified, efficient command system. That command system coordinates flexible, orderly management strategies in response to public health emergencies, such as COVID-19. Notable among those strategies have been the innovative measures of the Medical Laboratory Center (MLC) and Human Resources Center (HRC) during the epidemic.

The Luohu Hospital Group’s MLC, which was established in 2015, integrates the medical laboratories and resources that were previously operated separately by the group’s five hospitals. The MLC is a centralized, relatively independent medical laboratory that is responsible for all medical testing within the health-care system; it also offers third-party medical testing services to external institutions and the general public. With an ISO 15189 international standard laboratory certificate, the MLC has a 9,000-m^2^ laboratory and 122 employees. During the epidemic, the MLC set up a professional team specifically for COVID-19 nucleic acid testing. The team offers a 24/7 emergency response testing service; that service provides critical support for the timely screening of high-risk individuals and identifies infected community residents. Thanks to this integrated resource system, the MLC is able to prepare and accumulate test resources quickly in response to this emergency situation. Further, with its centralized service-delivery structure, the MLC has substantially improved its efficiency. The MLC initiated nucleic acid testing on January 29; subsequently, the daily detection capacity increased to 8000 cases/day in under 2 weeks. Construction of a standby polymerase chain reaction laboratory for COVID-19 detection was completed in less than 10 days; thereby, the daily nucleic acid testing capacity increased to 12,000 cases/day. At present, the MLC has the highest capacity for COVID-19 nucleic acid testing in Eastern China.

Established at the same time as the MLC, the HRC of the Luohu Hospital Group is responsible for human resources management of the entire integrated health-care system. The HRC is authorized to allocate human resources within the integrated health-care system in a timely manner. According to trends and changes in community health care as well as fluctuating patient numbers, the HRC is able to allocate medical staff from community health service institutions to hospitals. This structure is an important institutional basis for the rapid, flexible allocation of human resources for public health emergencies. For example, following regulatory requirements of the local government during the COVID-19 epidemic, the Luohu Hospital Group temporarily closed its outpatient services for stomatology and otolaryngology in addition to other hospital departments. The HRC was able to allocate medical staff from such departments to community health service institutions as well as to the operation department for the Internet Hospital (detailed below), which were short of manpower. In that way, the HRC was able to improve the efficiency of human resources management in the entire health-care system.

- **General practitioner team-based community containment strategy**

Since 2015, Luohu residents have been encouraged to sign a contract with a general practitioner based at a community health service institution. That represents their first point of contact with the Luohu Hospital Group. Supporting the general practitioner is a primary health-care team, which includes a nurse, a public health physician, and a health promotion practitioner. Such multidisciplinary teams play an important role in containing the community transmission of COVID-19 [8].

**Figure 2:**
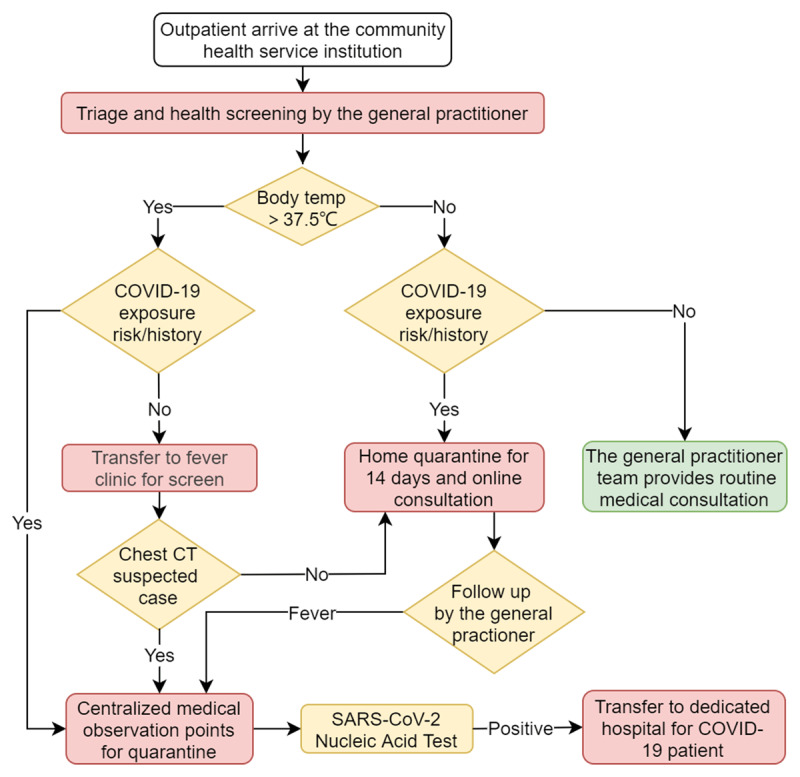
Flowchart of the general practitioner team–based COVID-19 community containment strategies in the Luohu Hospital Group.

The Luohu Hospital Group has set up 316 such general practitioner teams, which cover 527,000 contracted community residents. In the epidemic, the general practitioner teams have been responsible for the screening, triage, and health risk monitoring of residents. High-risk residents with an epidemic contact history are tracked and quarantined. All the epidemic control and prevention work in the community is conducted in accordance with standard operating procedures (***Figure 2***). In 2016, the Luohu Hospital Group conducted a pilot program for health promotion practitioners. Through cooperation with residents, the group recruited community workers to conduct professional health-care knowledge training. After being accredited by the group, those practitioners have joined the general practitioner teams to promote health and education among local residents. Presently, the system has 668 internally accredited health promotion practitioners. During the epidemic, those practitioners were responsible for COVID-19 epidemiological investigations as well as health monitoring and health education for residents quarantined at home. The practitioners have long worked in the community and are familiar with the situations that residents face. As a result, residents are able to achieve better compliance with health education interventions. It has also has been observed that the high-risk population in the community is more willing to cooperate with COVID-19 tracking, quarantine, and health monitoring when conducted by a health promotion practitioner.

The social distancing measures widely implemented during the epidemic may have led to psychological problems for residents. Based on the existing general practitioner hotlines, the Luohu Hospital Group provided psychological counseling services at an early stage of the epidemic. The HRC has allocated psychiatrists in the system to create a task force to provide residents with teleconsulting support. The group has improved risk communication with contracted residents through social and multiple media. To deal with anxiety, fatigue, and other health problems among medical staff resulting from the intensive work related to epidemic control and community prevention, the group provides necessary psychological crisis interventions and psychological counseling based on timely evaluation.

- **Synergizing e-health and telemedicine in health-care delivery**

Since 2015, the Luohu Hospital Group has undertaken two major actions to achieve integrated care: it has established integrated electronic medical record systems, and it has enhanced the interoperability of e-health across facilities and services. Those actions improved the group’s information systems; they permitted a quick response to patient behavior change during the epidemic following a surge in online medical consultations.

In April 2019, the Luohu Hospital Group set up its Internet Hospital; in February 2020, the Internet Hospital opened for service. It became an important platform of the integrated health-care system for telemedicine. The design and development of the Internet Hospital were based on the group’s integrated electronic health record system. That allows a hospital to access directly the health information about contracted residents. Via telemedicine, it provides the general practitioner teams with critical technical support in identifying high-risk patients in a timely manner. The Internet Hospital of the Luohu Hospital Group can offer various telemedical services to the public, such as the following: online consultations; electronic prescriptions; drug delivery; multidisciplinary team consultations; making an appointment for an examination; and online follow-up. Using the integrated medical system of the Internet Hospital, patients can access all their medical records at different institutions.

In response to the COVID-19 outbreak in Shenzhen, the Internet Hospital in March 2020 provided 9431 online consultations, 3837 electronic prescriptions, and 3822 drug deliveries to patients. There were 65,201 offline outpatient visits during that period; thus, online medical services had become an important supplement to offline ones. Through the Internet Hospital, telemedicine was able to meet the surging medical needs of local residents in emergencies; however, it also offered medical services for non-local patients. In March 2020, 17.84% of online consultations derived from patients outside Shenzhen. Following negotiations with the medical insurance bureau, the Luohu Hospital Group in April 2020 was able to obtain reimbursement through the medical insurance of patients with chronic diseases seeking online follow-up consultations and prescriptions. Such strategies have helped promote the transfer of patients from offline to online services, which avoids the physical gathering of patients. That diminishes the risk of infections in medical institutions and improves residents’ accessibility to medical services in public health emergencies.

## CONCLUSION

Establishing an integrated health-care system depends on the specific local context; there is a general lack of knowledge about how to respond to pandemics and other public health emergencies. After the first person-to-person transmission of COVID-19 was reported in a familial cluster in Shenzhen [9] and a retrospective study demonstrated that isolation and contact tracing could reduce the period when cases are infectious in the community [10], the Luohu Hospital Group undertook the effective efforts presented in this paper. In September 2017, the Luohu model was acknowledged and applied to people-centered integrated care in the whole of China by the country’s National Health Commission [11]. The successes of the response strategies of integrated health-care systems based on the Luohu model may provide a reference both for other city health systems in China and for other parts of the world in dealing with the pandemic.

To improve the public health emergency response capacity, it is necessary to enhance the public health workforce in primary health care; it is also essential to integrate resources to allow for regional sharing and efficient use, which would be easy to practice and benefit from the integrated health-care system. During the COVID-19 epidemic, the community containment strategy in the Luohu Hospital Group (based on general practitioner teams and employing e-health and telemedicine) facilitated the transformation of health-care delivery from a general to an emergency situation. Cooperation and joint efforts within an integrated health-care system help a community respond better to a public health emergency. Risk communication and community engagement are priority actions and deserve greater attention for the next stage in developing an integrated health-care system [12].

## DATA AVAILABILITY STATEMENT

All data relevant to the study are included in the article.

## References

[1] Zhang H. Early lessons from the frontline of the 2019-nCoV outbreak. The Lancet. 2020; 395: 687 DOI: 10.1016/S0140-6736(20)30356-1 PMC7135082 32059798

[2] Li X, Krumholz HM, Yip W, et al. Quality of primary health care in China: challenges and recommendations. The Lancet. 2020; 395: 1802–1812. DOI: 10.1016/S0140-6736(20)30122-7 PMC7272159 32505251

[3] World Bank and World Health Organization. Healthy China: Deepening Health Reform in China: Building High-Quality and Value-Based Service Delivery Washington, DC: World Bank Publications 2019 DOI: 10.1596/978-1-4648-1263-7

[4] Viktoria SK, Barbazza ES, Tello J, et al. Towards people-centred health services delivery: a Framework for Action for the World Health Organisation (WHO) European Region. International Journal of Integrated Care. 2013; 13: e058 DOI: 10.5334/ijic.1514 24409110 PMC3886596

[5] Wang X, Sun X, Birch S, et al. People-centred integrated care in urban China. Bulletin of the World Health Organization. 2018; 96: 843–852. DOI: 10.2471/BLT.18.214908 30505032 PMC6249708

[6] Ballard M, Bancroft E, Nesbit J, et al. Prioritising the role of community health workers in the COVID-19 response. BMJ Global Health. 2020; 5: e002550 DOI: 10.1136/bmjgh-2020-002550 PMC7298684 32503889

[7] Rowe SY, Rowe AK, Peters DH, et al. Effectiveness of strategies to improve health-care provider practices in low-income and middle-income countries: a systematic review. The Lancet Global Health. 2018; 6: e1163–e1175. DOI: 10.1016/S2214-109X(18)30398-X 30309799 PMC6185992

[8] Zhang X, Zhou H, Zhang W, et al. Assessment of Coronavirus Disease 2019 Community Containment Strategies in Shenzhen, China. JAMA Network Open. 2020; 3: e2012934 DOI: 10.1001/jamanetworkopen.2020.12934 32568401 PMC7309437

[9] Chan JF, Yuan S, Kok K, et al. A familial cluster of pneumonia associated with the 2019 novel coronavirus indicating person-to-person transmission: a study of a family cluster. The Lancet. 2020; 395: 514–523. DOI: 10.1016/S0140-6736(20)30154-9 PMC7159286 31986261

[10] Bi Q, Wu Y, Mei S, et al. Epidemiology and transmission of COVID-19 in 391 cases and 1286 of their close contacts in Shenzhen, China: a retrospective cohort study. The Lancet Infectious Diseases. 2020 DOI: 10.1016/S1473-3099(20)30287-5 PMC7185944 32353347

[11] Wang X, Sun X, Gong F, et al. The Luohu Model: A Template for Integrated Urban Healthcare Systems in China. International Journal of Integrated Care. 2018; 18 DOI: 10.5334/ijic.3955 30483036 PMC6199563

[12] Hu G, Qiu W. From guidance to practice: Promoting risk communication and community engagement for prevention and control of coronavirus disease (COVID-19) outbreak in China. Journal of Evidence-Based Medicine. 2020; 13: 168–172. DOI: 10.1111/jebm.12387 32445287 PMC7280730

